# Valedictory

**Published:** 1874-11

**Authors:** Wm. Abram Love, V. H. Taliaferro


VALEDICTORY.—It will be seen that with the present number, our connection with the JOURNAL, as associate editors, terminates. In withdrawing, we desire to state that the rapidly increasing prosperity of the JOURNAL for the past twelve months has been due to the energy and untiring labors of Dr. Battey, the editor and business manager.

In taking our leave, we do so feeling an unabated interest in its continued prosperity. And while we gladly take position again in the rank of contributors, we shall not relax our efforts at all in its behalf.

WM. ABRAM LOVE, M.D.
V. H. TALIAFERRO, M.D.

